# Quantitative analysis of gastrointestinal fluid absorption and secretion to estimate luminal fluid dynamics in rats

**DOI:** 10.1038/s41598-023-44742-y

**Published:** 2023-10-14

**Authors:** Yuta Funai, Kazuki Ichijo, Satoru Suzuki, Yuta Tateishi, Katsuhisa Inoue, Ikumi Tamai, Yoshiyuki Shirasaka

**Affiliations:** 1 Faculty of Pharmacy, Institute of Medical, Pharmaceutical and Health Sciences, Kanazawa University, Kakuma-Machi, Kanazawa, Ishikawa 920-1192 Japan; 2 School of Pharmacy, Tokyo University of Pharmacy and Life Sciences, 1432-1 Horinouchi, Hachioji, Tokyo 192-0392 Japan

**Keywords:** Drug discovery, Physiology

## Abstract

The drug absorption profile is dependent on the luminal drug concentration, which in turn is influenced by the gastrointestinal (GI) fluid dynamics. In the present study, therefore, we aimed to examine the luminal fluid dynamics by kinetically analyzing fluid absorption and secretion along the GI tract in rats using the in situ closed-loop technique with non-absorbable fluorescein isothiocyanate-dextran 4000 (FD-4) and tritium water labeling ([^3^H]water) under different osmotic conditions. We found that the luminal fluid volume in the jejunum and ileum, but not the colon, gradually decreased and reached a steady state. In contrast, [^3^H]water almost completely disappeared in all intestinal regions. Kinetic analysis revealed the following rank order for the rate constant of fluid secretion: jejunum > ileum > colon, whereas a negligible regional difference was observed in the rate constant of fluid absorption. Fluid secretion under an isosmotic condition (300 mOsm/kg) was higher than that at 0 mOsm/kg in all intestinal regions, though no significant changes in fluid absorption were observed. Thus, the fluid secretion process appears to be the major determinant of the regional differences in GI fluid dynamics. Our findings indicate that the luminal fluid volume is altered as a result of water ingestion, absorption, and secretion, and finally reaches an apparent steady state, which is regulated mainly by the process of fluid secretion.

## Introduction

Gastrointestinal (GI) absorption of drugs in solution occurs via both passive diffusion and active transport via drug transporters, and, in addition, may be influenced by metabolic enzymes. Generally, GI absorption of a drug through these mechanisms depends upon the concentration gradient across the membrane and/or the solubility of the drug. Based on the Biopharmaceutical Classification System (BCS), when a drug has low solubility but high permeability (BCS class II), dissolution becomes the rate-limiting step in drug absorption, while when a drug has high solubility but low permeability (BCS Class III), permeation becomes the rate-limiting step. To understand the GI absorption of drugs, including these concepts of dissolution and permeation, it is important to know the drug concentrations in various parts of the GI tract and the relevant variables. Fluid volume is a major factor directly determining GI drug concentration and the amount of dissolution of poorly soluble drugs in the GI tract. We recently reported that changes in the GI fluid volume may indirectly influence the drug absorption profile by altering the drug concentration and absorption kinetics^1–5^. Furthermore, we and other research groups demonstrated that water secretion and drug resultant dilution of drug concentration in the GI tract may contribute to beverage-drug interactions that result in decreased drug absorption^1,6,7^. Thus, it has been noted that GI fluid dynamics concepts to date are not sufficient for the development of oral drug delivery and prediction of oral drug absorption^8^.

GI fluid volume in humans (including children and adults) and rats has been investigated by means of magnetic resonance imaging (MRI) and positron emission tomography (PET)^6,8–11^. However, GI fluid dynamics is complicated by multiple factors such as fluid flux across the intestinal membrane, GI transit, gastric emptying rate, and physiological environment (pH, sodium concentration, and carbohydrate, secretions such as salivary, gastric, gall bladder, and pancreatic secretions)^12^. Therefore, GI fluid dynamics observed by MRI and PET may include the effects of these multiple factors, implying apparent fluid dynamics in the GI tract. On the other hand, real fluid dynamics (fluid flux across the intestinal membranes) remains unclear. This may also have regional differences in fluid absorption and secretion (e.g., jejunum, ileum, and colon). Thus, in order to clarify the real fluid absorption and secretion, it is necessary to separate the apparent fluid dynamics from the real fluid dynamics.

The fluid volumes in the stomach and small intestine have been measured, but data on the fluid volume and its variation in the colon are still scarce^13,14^. Murray et al. reported colonic fluid volume after water administration in humans^14^. They also reported colonic fluid volume after administration of hyperosmotic solutions such as carbohydrate solutions and fruit juices^15^. However, it is not easy to compare such fluid dynamics in the colon with those in the small intestine in terms of fluid flux across intestinal membranes. It is well-known that the function of the colon is to absorb water, keep the fluid volume low, and form feces, and fluid movement in the colon is of great pathophysiological importance in relation to diarrhea and constipation. On the other hand, since the major site of drug absorption is not the colon, but the small intestine, it seems that fluid dynamics in the small intestine is more important than that in the colon in determining the absorption and pharmacokinetics of oral drugs. Nevertheless, the colon can be an important site of drug absorption from some controlled-release formulations (e.g., sustained-release and slow-release formulations) and thus must sometimes be considered during the development of drug delivery systems^16^. Therefore, an accurate assessment of fluid dynamics throughout the GI tract is necessary to predict GI drug concentrations and absorption kinetics.

Several physiologically based pharmacokinetic (PBPK) models for predicting oral drug absorption (e.g., the advanced compartmental absorption and transit (ACAT) and the advanced dissolution, absorption, and metabolism (ADAM) models, which are also included in commercially available PBPK modeling software) have already incorporated fluid dynamics^17–19^. However, because fluid flux across the membrane remains unclear, these models and related studies employ apparent fluid dynamics, and do not reflect real fluid absorption and secretion behavior. Also, reports on GI fluid dynamics include both apparent and real fluid dynamics due to differences in experimental methods. In the present study, we aimed to clearly separate and analyze fluid absorption and secretion along the GI tract in rats.

## Results

### Regional differences of apparent fluid absorption in rat intestine

**Figure 1:**
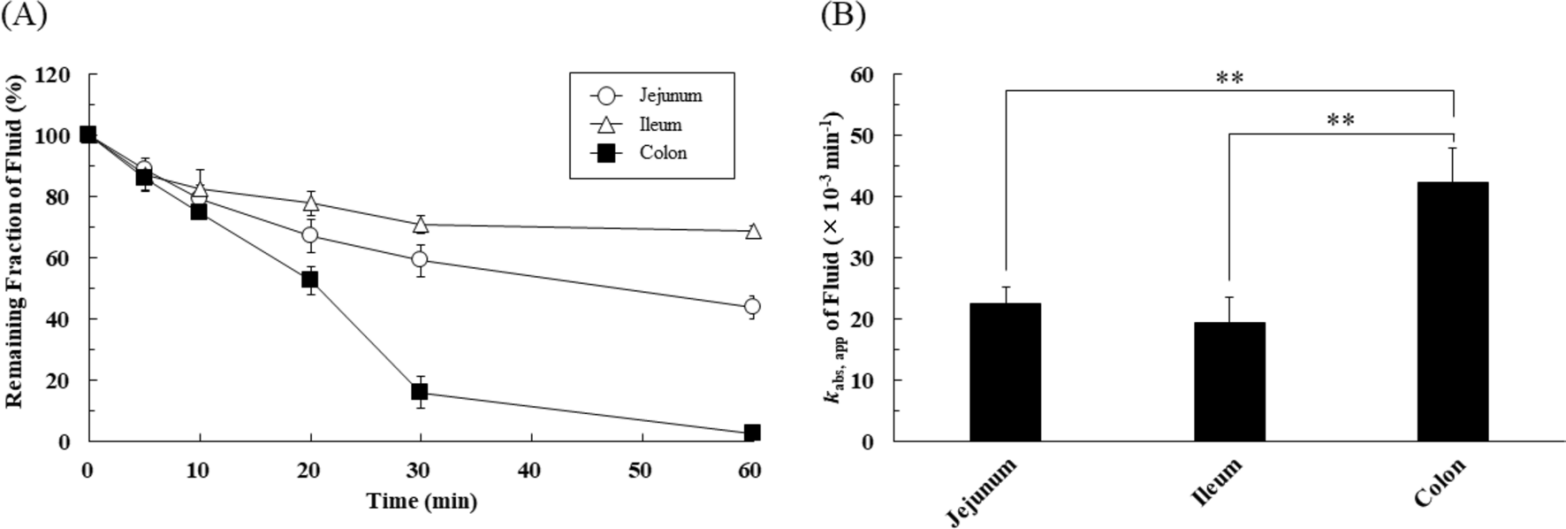
Regional differences in apparent fluid absorption in rat intestine. (**A**) The remaining fraction of fluid at 5, 10, 20, 30, and 60 min in the jejunum (open circle), ileum (open triangle), and colon (closed square). Results are expressed as mean ± SEM (n = 3–8 rats at each time point). A total of 27 rats were used for the in situ closed-loop studies. (**B**) The apparent fluid absorption rate constant (*k*_abs,app_) in each region was determined by the in situ closed loop method using FD-4 (10 μM, 1 mL). Results are expressed as mean ± SEM. Statistical significance was calculated by one-way ANOVA with Tukey’s multiple comparisons test (**p < 0.01).

The regional differences of GI fluid absorption were evaluated by monitoring the time courses of the remaining fraction of fluid in the rat jejunum, ileum, and colon after administration of fluorescein isothiocyanate-dextran 4000 (FD-4) using an in situ closed-loop method. As shown in Fig. 1A, the remaining fraction of fluid gradually decreased and appeared to reach an apparent steady-state after 30 min. Since fluid absorption showed first-order kinetics for at least up to 30 min, the apparent absorption rate constants (*k*_abs,app_) of fluid were calculated from the data up to 30 min (Fig. 1B). The values of *k*_abs,app_ in the jejunum, ileum, and colon are 21.4 ± 2.7 × 10^–3^ min^-1^ (n = 17), 17.2 ± 4.1 × 10^–3^ min^-1^ (n = 21), and 42.3 ± 5.8 × 10^–3^ min^-1^ (n = 19), respectively. The *k*_abs,app_ of fluid was significantly higher in the colon than in the jejunum or ileum, whereas it was similar in the jejunum and ileum.

### Regional differences of real fluid absorption in rat intestine

**Figure 2:**
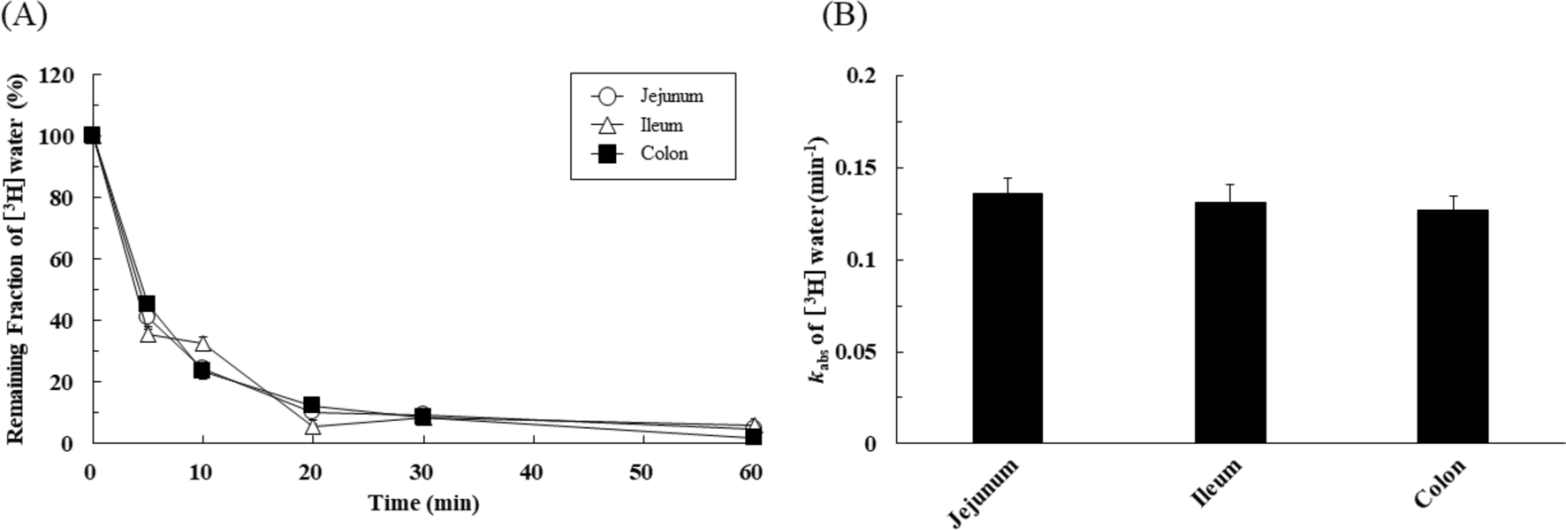
Regional differences in real fluid absorption in rat intestine. (**A**) The remaining fraction of [^3^H]water at 5, 10, 20, 30, and 60 min in the jejunum (open circle), ileum (open triangle), and colon (closed square). Results are expressed as mean ± SEM (n = 5–10 rats at each time point). A total of 36 rats were used for the in situ closed-loop studies. (**B**) the real fluid absorption rate constant (*k*_abs_) in each region were determined by the in situ closed-loop method using [^3^H]water (dosage; 1 μCi/mL, 1 mL). Results are expressed as mean ± SEM. Statistical significance calculated by one-way ANOVA with Tukey’s multiple comparisons test.

Theoretically, intestinal fluid volume is defined as the sum of the absorption of ingested water and biological fluid and the fluid secretion from the body. Therefore, it is considered that the time profiles of intestinal fluid volume shown in Fig. 1A represent the apparent fluid dynamics in the intestine. To monitor real fluid absorption, the time courses of the remaining fraction of applied water itself were next evaluated by using [^3^H]water. Interestingly, rapid water absorption was observed in all intestinal regions (Fig. 2A). The absorption rate constant (*k*_abs_) of [^3^H]water, that is the real fluid absorption rate constant, was estimated from the data up to 30 min. As shown in Fig. 2B, the values of *k*_abs_ in the jejunum, ileum, and colon were 0.136 ± 0.009 min^-1^ (n = 31), 0.131 ± 0.010 min^-1^ (n = 28), and 0.127 ± 0.008 min^-1^ (n = 23), respectively, and there was no significant difference, in contrast to the case of *k*_abs.app_ (Fig. 1B).

### Regional differences in fluid secretion in rat intestine

**Figure 3:**
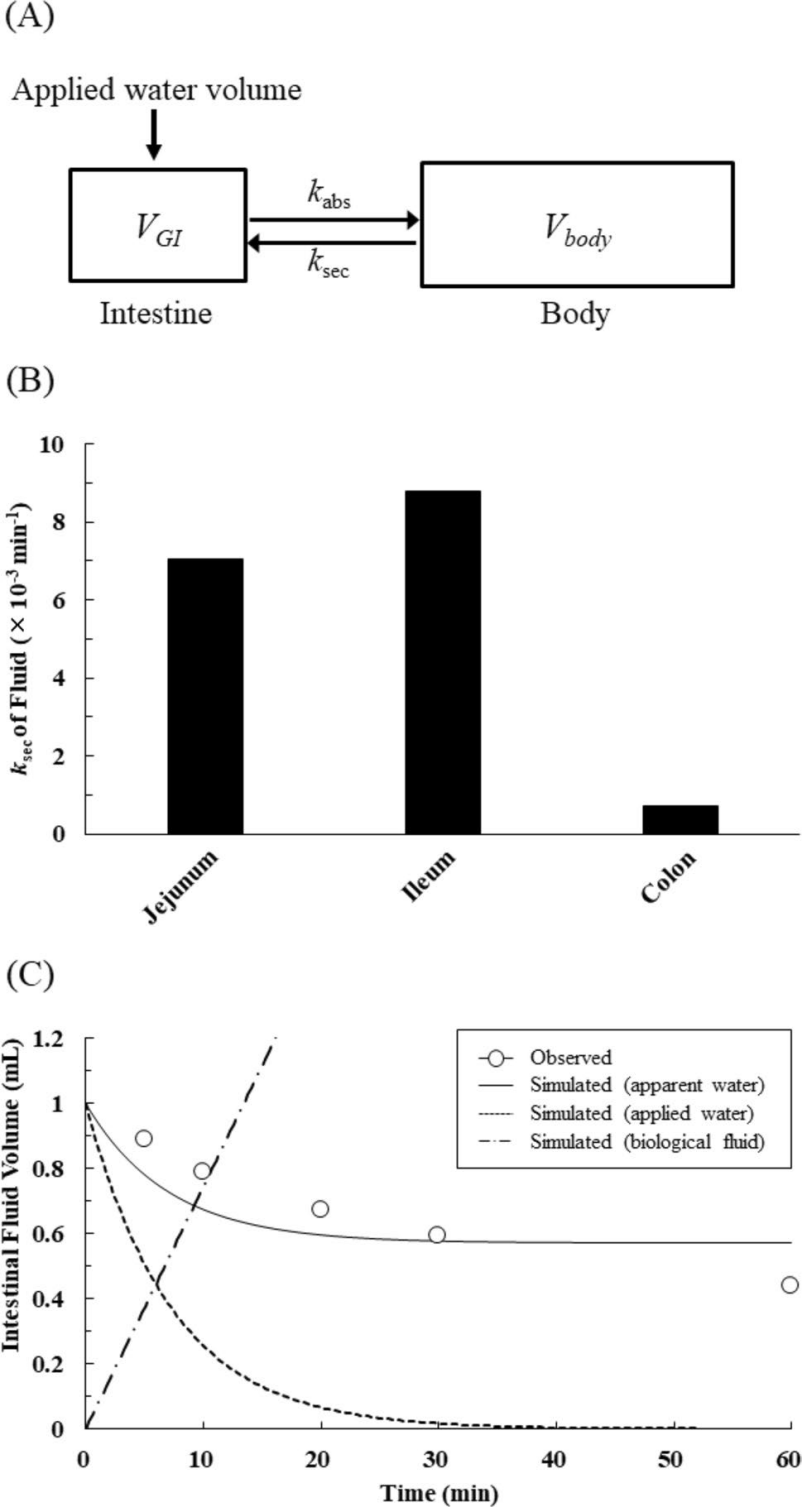
Regional differences in fluid secretion in rat intestine. (**A**) The simple kinetic model was used to determine the rate constant of fluid secretion (*k*_sec_) in the rat intestine. The applied water volume is 1 mL (the initial dosing volume in in situ closed-loop experiments). *V*_GI_ and *V*_body_ represent the volume of fluid in each region of the GI tract and the total body fluid volume, respectively. The rate constant of fluid absorption (*k*_abs_) is obtained from Fig. 2. (**B**) The values of the rate constant of fluid secretion (*k*_sec_) in jejunum, ileum, and colon were calculated by fitting analysis using the simple kinetic model shown. (C) Simulation of the time profiles of absorption of ingested water, secretion of biological fluid, and apparent fluid dynamics in rat jejunum. The data are taken from Fig. 1A.

To estimate the rate constant of fluid secretion (*k*_sec_), the time courses of the remaining fraction of fluid (Fig. 1A) were fitted to a simple kinetic model (Fig. 3A) with the *k*_abs_ values obtained from Fig. 2B. The values of *k*_sec_ of fluid in the jejunum, ileum, and colon were 7.05 × 10^–3^ min^-1^, 8.78 × 10^–3^ min^-1^, and 0.710 × 10^–3^ min^-1^, respectively (Fig. 3B). The value in the colon was significantly lower than in the small intestine, whereas the values were similar in the jejunum and ileum. Figure 3C shows the results of simulation of the time profiles of absorption of ingested water, secretion of biological fluid, and apparent fluid dynamics in the jejunum (as an example) based on the fitting analysis performed in Fig. 3B. The results support the idea that intestinal fluid dynamics is influenced by multiple factors, including the absorption of ingested water and biological fluid and fluid secretion from the body.

### Effect of solution osmolality on intestinal fluid dynamics in rat

**Figure 4:**
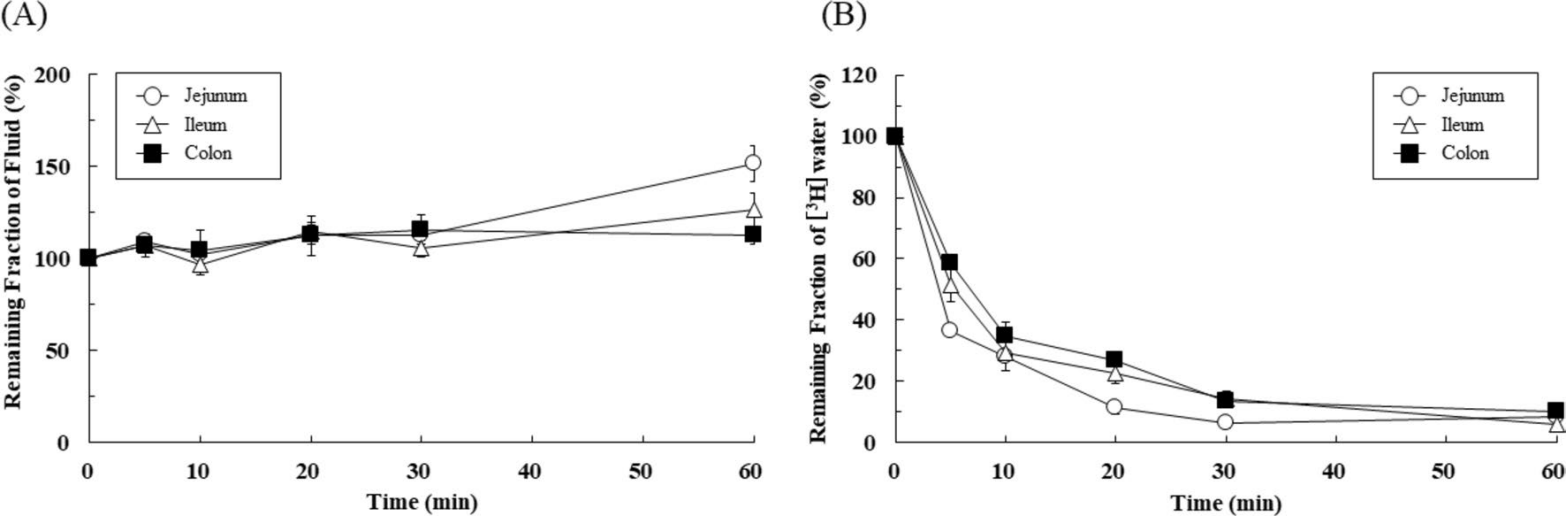
Effect of solution osmolality on fluid absorption in rat intestine. The remaining fractions of (**A**) water and (B) [^3^H]water at 5, 10, 20, 30, and 60 min in jejunum (open circle), ileum (open triangle), and colon (closed square) were determined by the in situ closed-loop method using FD-4 (10 μM, 1 mL) and [^3^H]water (1 μCi/mL, 1 mL), respectively, in mannitol solution (300 mOsm/kg). Results are expressed as mean ± SEM ((**A**) n = 5–10 rats and (**B**) 3–8 rats at each time point). A total of (**A**) 27 rats and (**B**) 27 rats were used for the in situ closed-loop studies.

The effects of solution osmolality on fluid absorption and secretion in the rat intestine (jejunum, ileum, and colon) were investigated using in situ closed-loop method. Apparent fluid absorption and real fluid absorption after administration of mannitol solution (300 mOsm/kg) were evaluated using FD-4 and [^3^H]water, respectively (Fig. 4). As shown in Fig. 4A, no change in the apparent fluid volume was observed in any intestinal region, presumably due to the isosmotic conditions. In contrast, real fluid absorption was rapid, regardless of isosmotic conditions (Fig. 4B).

**Figure 5:**
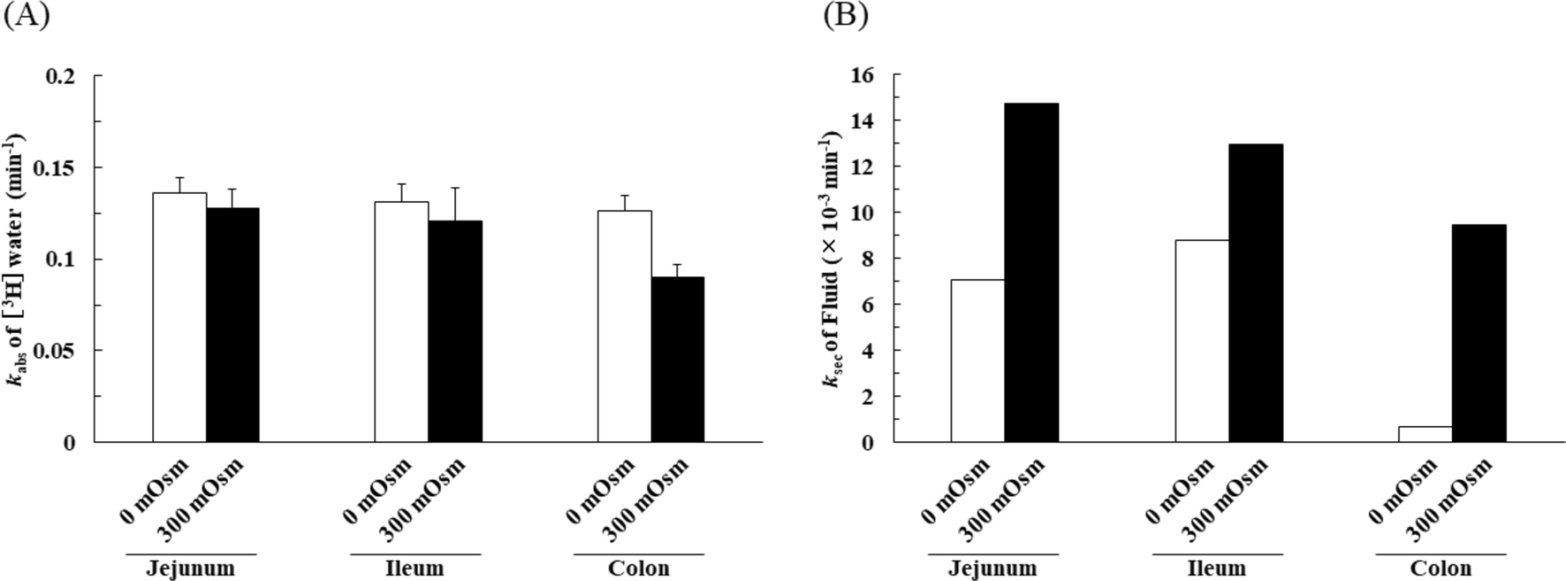
Regional differences in the effect of solution osmolality on the rate constants of fluid absorption and secretion in rat intestine. (**A**) The real fluid absorption rate constant (*k*_abs_) and (**B**) the fluid secretion rate constant (*k*_sec_) in each region were determined based on the results in Fig. 5. The data obtained at 0 mOsm/kg condition (purified water) are reused from Figs. 3B and 4A. Results are expressed as mean ± SEM. In Fig. 5A, no statistical significance between 0 mOsm/kg and 300 mOsm/kg was calculated by one-way ANOVA with Tukey’s multiple comparisons test.

When these results were analyzed using a kinetic model (Fig. 3A), the values of the absorption rate constant (*k*_abs_) of [^3^H]water in mannitol solution (300 mOsm/kg) were estimated to be 0.128 ± 0.011 min^-1^ (jejunum), 0.121 ± 0.018 min^-1^ (ileum), and 0.0900 ± 0.00703 min^-1^ (colon) (Fig. 5A). The values of *k*_sec_ of fluid in mannitol solution (300 mOsm/kg) were 14.8 × 10^–3^ min^-1^ (jejunum), 13.0 × 10^–3^ min^-1^ (ileum), and 9.45 × 10^–3^ min^-1^ (colon), respectively (Fig. 5B). Overall, there was no significant difference between the *k*_abs_ values of fluid between 0 mOsm/kg and 300 mOsm/kg solutions in all intestinal regions, whereas the *k*_sec_ value was markedly higher at 300 mOsm/kg than at 0 mOsm/kg.

## Discussion

Gastrointestinal fluid volume has an important influence on drug absorption profiles defined in terms of drug concentration, and in recent years, GI fluid dynamics has attracted increasing attention. Indeed, dynamic variations in fluid volumes along the GI tract have been observed by means of fluid-sensitive MRI and PET analyses in healthy volunteers and/or experimental animals^6,11^. Furthermore, our recent studies have shown that the solution osmolality influences the GI fluid volume^1–5^. We also demonstrated that osmolality-dependent variations in GI fluid volume can alter drug concentration in the GI tract, and consequently change drug absorption characteristics^2^. This could be one of the factors underlying beverage-drug interactions^1,6,8^. However, when ingested substances (including food and beverage components) are absorbed, the effect of osmolality on fluid dynamics will be complicated. Therefore, it is important to understand the basics of the fluid environment involved in pharmacokinetics, and quantitative analysis of GI fluid dynamics provides valuable information not only for pharmacokinetics but also for GI physiology and pharmacometrics. In the present study, we aimed to quantitatively examine the luminal water dynamics by kinetically analyzing fluid absorption and secretion along the GI tract in rats.

In Fig. 1, the intestinal fluid volume obtained from experiments with FD-4 showed an exponential decrease, suggesting first-order fluid absorption up to at least 30 min. The *k*_abs,app_ value of fluid is higher in the colon than in the small intestine (jejunum and ileum). Interestingly, luminal fluid volume reached a steady state within 30–60 min in a site-dependent manner. A similar tendency has been observed after the oral administration of water in humans^9,14^. However, the luminal fluid volume is regulated not only by the fluid absorption process but also by the secretion of biological fluid. Thus, the results (*k*_abs,app_ value) in Fig. 1 do not represent the real fluid dynamics, but rather the apparent fluid dynamics. Therefore, we next tried to evaluate real fluid absorption in the rat GI tract by using [^3^H]water (Fig. 2). Interestingly, [^3^H]water disappeared rapidly after administration, and the real fluid absorption rate constant (*k*_abs_) showed no significant difference among regions, unlike the *k*_abs,app_ values (Figs. 1B and 2B). On the other hand, the *k*_sec_ values were markedly lower in the colon than in the small intestine (jejunum and ileum) (Fig. 3B). These results indicate that the apparent site-dependent intestinal fluid flux across the membrane observed in Fig. 1 mainly reflects the fluid secretion process. It is widely known that the function of the colon is to absorb water, keep the fluid level low, and form feces, and it has been assumed that the fluid absorption capacity of the colon is higher than that of the small intestine. In contrast, our findings show that there is no regional difference in fluid absorption capacity, and that the low fluid environment in the colon is due to low fluid secretion capacity. The mechanism of the regional differences in fluid secretion is unclear, but we are currently studying the molecular mechanisms of fluid absorption and secretion in the GI tract, focusing on the molecular species of the water channels (aquaporin, AQP) and mucin (MUC), and their expression levels and functions. Nevertheless, our present results indicate that the fluid secretion process is the major determinant of GI fluid dynamics and its regional differences. We recently reported that the GI fluid volume after administration of different volumes of purified water gradually decreased to a similar steady-state level^9^. Together with the present results, this supports the idea that the steady-state level of fluid in the GI tract is not affected by the administered fluid volume, but is defined by the homeostatic biological fluid secretion as well as fluid absorption processes. On the other hand, biological fluid secretion is not only coming from intestinal walls but also from salivary and gastric secretions and subsequent gastric emptying as well as gall bladder and pancreatic secretions. The influence of these various types of secretions would also be very important in considering GI fluid dynamics. The in situ closed-loop method used in this study can exclude the influence of other physiological secretions and their transit processes. Further studies, including in vivo experiments, would be needed to consider the effects of various secretions on GI fluid dynamics.

A previous study with D_2_O or [^3^H]water found that fluid absorption in the GI tract is dependent on blood flow^20^. This is consistent with our finding that there is no significant regional difference in *k*_abs_. Namely, since real fluid absorption in the GI tract is extremely rapid and is rate-limited by the blood flow, no regional differences in fluid absorption would be observed even if regional differences exist in the real fluid absorption rate. It has been reported that intestinal absorption of some drugs such as antipyrine and theophylline is rate-limited by blood flow^21–23^. As shown in Table 1, the permeability of fluid was estimated to be similar to that of antipyrine, suggesting that the rate-limiting step for fluid absorption is indeed blood flow. In addition, we next attempted to directly compare fluid absorption clearance and blood flow. The effective mucosal blood flow involved in fluid absorption is considered to be blood flow at the lining of the villi through the epithelial cells (villous blood flow, *Q*_villi_)^24,25^. Since it was reported that *Q*_villi_ in humans represents about 20% of the portal blood flow (*Q*_portal_) and *Q*_portal_ in rats is 32.9 mL/min/kg, *Q*_villi_ is estimated to be 6.58 mL/min/kg (= 0.2 × 32.9 mL/min/kg), assuming no species difference between human and rat^26,27^. Then, the *Q*_villi_ in each intestinal region used in this experiment can be calculated to be 0.116 mL/min on the assumption that *Q*_villi_ is proportional to the length of each region (10 cm [for experiments]/142 cm [total intestine]^28,29^). In this case, calculated *CL*_abs_ is very similar to *Q*_villi_, further supporting the idea that the rate-limiting step for fluid absorption is blood flow. On the other hand, in the in situ study, anesthesia could alter blood flow, which might affect fluid dynamics in the GI tract. The similarity between *CL*_abs_ (obtained under anesthesia) and *Q*_villi_ (obtained under non-anesthesia) suggests that anesthesia may not have a significant effect on fluid dynamics (*k*_abs_ and *k*_sec_ values). Further analysis of anesthetic effects on blood flow and fluid dynamics is warranted.

**Table 1:** Gastrointestinal permeability of fluid in rats.

Intestinal region	Water ([^3^H]water)		Antipyrine^35^
	*CL*_abs_(mL/min)	*P*_eff_(× 10^–4^ cm/sec)	*P*_eff_(× 10^–4^ cm/sec)
Jejunum	0.136	2.02	1.50
Ileum	0.131	1.95	1.12
Colon	0.127	1.89	1.08

The absorption clearance (*CL*_abs_) and membrane permeability (*P*_eff_) in rat intestines are calculated from Eq. (4) and the *k*_abs_ of fluid in Fig. 2.

Theoretically, GI fluid movement is dependent upon luminal osmolality. For example, it can be inferred that the fluid in the GI lumen apparently shows very little movement when isosmotic solutions are administered. As shown in Fig. 4A, no change in the apparent fluid volume was observed using FD-4, presumably due to the isosmotic (300 mOsm/kg) condition. However, in the case of [^3^H]water, rapid absorption was observed, regardless of isosmotic condition, suggesting that osmotic pressure has no influence on real fluid absorption (Fig. 4B). Very interestingly, no significant difference in the *k*_abs_ value of fluid was observed between 0 mOsm/kg and 300 mOsm/kg solutions in any intestinal region, whereas the *k*_sec_ value was markedly higher at 300 mOsm/kg condition than at 0 mOsm/kg (Fig. 5). These findings strongly support our hypotheses that (1) the fluid absorption process is rate-limited by the blood flow and (2) the fluid secretion process is the major determinant of fluid flux across the intestinal membrane and its regional differences in GI tract.

Figure 5A seems to show that the *k*_abs_ value of fluid in the colon was decreased under 300 mOsm/kg condition compared to 0 mOsm/kg condition, although the difference did not reach statistical significance. Because the colon is known to have a smaller surface area and tighter junctions compared to the small intestine, the real permeability of fluid may be less in the colon than in the small intestine^30^. In this case, it is possible that the real fluid absorption clearance at 300 mOsm/kg is smaller than *Q*_villi_, resulting in a shift in the rate-limiting step from blood flow to permeation. In other words, the *k*_abs_ of fluid under hyperosmotic conditions (> 300 mOsm/kg) may not be rate-limited by blood flow. Predictions of GI fluid movement and its effect on oral drug absorption after ingestion of hyperosmotic solution, beverages, and food may need to take account of these possibilities.

In recent years, some studies dealing with GI fluid dynamics have not adequately distinguished real fluid movement from apparent fluid movement. The effect of gastric emptying of ingested water and gastric fluid on drug absorption was reported by Grimm et al., while many aspects of intestinal fluid dynamics remain unclear^31,32^. In the present study, we analyzed GI fluid dynamics quantitatively from both perspectives (apparent and real) using FD-4 and [^3^H]water. Our results indicate that real fluid absorption is independent of GI regions and osmolality, but is rate-limited by blood flow (although the colon may not, as mentioned above). Furthermore, the fluid secretion process is the major determinant of fluid flux across the membrane and its regional differences in GI tract. The use of apparent fluid dynamics may be sufficient to predict drug absorption under normal conditions using PBPK models, including the ACAT and ADAM models. However, precise analysis of fluid dynamics should make it possible to predict GI fluid movement under the influence of food and beverages, disease states, and even GI toxicity (diarrhea or constipation) of drugs, over a wide range of environments and conditions.

## Materials and methods

### Experimental animals and ethical approval

Male Wistar rats weighing 230 g ± 10% (7 weeks old) were purchased from Tokyo Laboratory Animals Science Co., Ltd. (Tokyo, Japan), and were maintained in a climate-controlled breeding facility under 12-h automatic ambient light cycle control at the Tokyo University of Pharmacy and Life Sciences. Treatments of experimental animals and relevant protocols complied with the Regulations on Performing Animal Experiments established by the Committee of Animal Care and Welfare of Tokyo University of Pharmacy and Life Sciences (approval number: P19-08) and the ARRIVE guidelines. All animal experiments (a total of 150 rats) were performed in accordance with the relevant guidelines and regulations. Additionally, we carried out animal anesthesia and euthanasia in accordance with American Veterinary Medical Association (AVMA) Guidelines. Specifically, rats were anesthetized with a triple anesthetic combination (propofol, midazolam, and alfentanil) as intraperitoneal anesthesia and euthanized with an overdose of isoflurane as inhalation anesthesia. Propofol, midazolam, and alfentanil were purchased from Meiji Seika Pharma Co., Ltd. (Tokyo, Japan), FUJIFILM Wako Pure Chemical Corporation (Osaka, Japan), and Nippon Zenyaku Kogyo Co., Ltd. (Fukushima, Japan), respectively. Isoflurane was purchased from Pfizer Inc. (NY, USA).

### In situ intestinal closed loop experiments in rats

The fluid volume and drug concentration in the rat GI tract were determined using the in situ closed-loop methods described by Funai *et* *al*^1,7^. Before the experiments, rats were fasted for at least 12 h with free access to water. Anesthesia was induced with a triple anesthetic combination. The intestine was exposed through a midline abdominal incision and an intestinal loop (jejunum, 10 cm; ileum, 10 cm; colon, 7 cm) was made by cannulation into both ends of the jejunum, ileum, or colon with silicone tubing (i.d., 3 mm). Each intestinal segment was cleaned by flushing with saline (37 °C). One mL of FD-4 (10 µM, a non-permeable marker) dissolved in purified water or mannitol solution (300 mOsm/kg, as an isotonic solution with an osmolality similar to blood and body fluids) or 1 mL of [^3^H]water (1 μCi/mL) dissolved in purified water or mannitol solution was introduced into the intestinal loop, and both ends of the loop were ligated to determine the impact of applied fluid volume on the remaining fraction of fluid in the rats. FD-4 was purchased from Sigma-Aldrich Company (St. Louis, MO). [^3^H]Water was purchased from PerkinElmer Life Sciences (Boston, Massachusetts, USA). The osmolality was measured with a cryoscopic osmometer, OSMOMAT 030-D (Gonotec GmbH, Berlin, Germany).

In experiments using FD-4 solution, the test solution in the loop was collected after 5, 10, 20, 30, or 60 min by flushing with air to measure the luminal concentration of FD-4, *C*_out,FD-4_, and then made up to 10 mL with buffer solution to measure the amount of FD-4, *X*_out,FD-4_. Assuming that FD-4 is homogeneously distributed in the lumen after intestinal administration, the fluid volume in the intestine (mL) was calculated using the following equation:

1 $$V_{{{\text{water}},{\text{app}}}} = X_{{{\text{out}},{\text{FD}} - {4}}} /C_{{{\text{out}},{\text{FD}} - {4}}}$$

where *V*_water,app_ (mL) is the calculated fluid volume in the intestine. *X*_out,FD-4_ is the amount of FD-4 (μmol), and *C*_out,FD-4_ is the luminal concentration of FD-4 (μM). The *V*_app,water_ was normalized to the surface area (11.2 cm^2^) calculated from the radius and length of the intestine reported by Komiya *et* *al*^33^*.* To verify the validity of the calculated *V*_water,app_ values, the volume of the withdrawn samples was routinely confirmed by directly weighing the samples. In addition, if the samples contained blood or the length of the intestinal loop used was not adequate, those rats were excluded from the analysis. To assure sampling accuracy, it has been confirmed that the recovery of FD-4 is more than 80% in all experiments. The concentration of FD-4 was measured using a microplate fluorescence reader (Varioskan™ Flash 2.4; Thermo Fisher Scientific Inc., Kanagawa, Japan) at excitation and emission wavelengths of 492 and 515 nm, respectively.

In experiments using [^3^H]water, the test solution in the loop was collected at the specified time points by flushing, and then made up to 10 mL with saline to measure the radioactivity of remaining [^3^H]water. Assuming that ^3^H-labeled and non-labeled water behave similarly in the lumen after intestinal administration, the concentration of [^3^H]water in purified water is always constant (1 μCi/mL, the initial concentration), and the remaining administered water volume in the intestine (mL) was calculated using the following equation:

2 $$V_{{{\text{water}}}} = X_{{{\text{out}},\left[ {{\text{3H}}} \right]{\text{water}}}} /C_{{{\text{out}},\left[ {{\text{3H}}} \right]{\text{water}}}}$$

where *V*_water_ (mL) is the calculated remaining fluid volume of administered water in the intestine. *X*_out,[3H]water_ is the amount of radioactivity of [^3^H]water (μCi), and *C*_out,[3H]water_ is the concentration of radioactivity of [^3^H]water (1 μCi/mL). The *V*_water_ was normalized to the surface area. Radioactivity of [^3^H]water was measured with a liquid scintillation counter (LSC-6100, Aloka, Tokyo, Japan).

### Determination of rate constants of intestinal fluid absorption and secretion

The rate constant of apparent fluid absorption (*k*_abs,app_) was determined using log-linear regression of the data at time points up to 30 min in the time course of the remaining fraction of fluid calculated using FD-4. Also, the real fluid absorption rate constant (*k*_abs_) was determined using log-linear regression of the data points at time points up to 30 min in the time course of the remaining fraction of fluid calculated using [^3^H]water. In addition, the population pharmacokinetics and Bayesian estimation were used to calculate the mean and SEM of the *k*_abs_ of fluid. The real fluid secretion rate constant (*k*_sec_) was determined using the simple rat GI kinetic model shown in Fig. 3A, according to the following equation:

3 $$dV_{{{\text{GI}}}} /dt = - k_{{{\text{abs}}}} \cdot \, V_{{{\text{GI}}}} + k_{{{\text{sec}}}} \cdot \, V_{{{\text{body}}}} \cdot\left( {S/S_{{{\text{sum}}}} } \right)$$

where *V*_GI_ is the time course of the remaining fraction of fluid calculated using FD-4 *V*_body_ is the total body fluid volume (initial condition: 167 mL)^34^, and *S* and *S*_sum_ are the intestinal surface area under the conditions of the in situ closed-loop experiment and the intestinal total surface area, respectively. The *k*_abs_ values of jejunum, ileum, and colon were those determined above (0.136, 0.131, and 0.127, respectively). The value of *k*_sec_ was optimized in the fitting step using this model.

Permeability (*P*_eff_) of fluid was calculated by the following equation:

4 $$P_{{{\text{eff}}}} = CL_{{{\text{abs}}}} /S = k_{{{\text{abs}}}} \times V_{{{\text{lumen}}}} /S$$

where *CL*_abs_ is the absorption clearance of fluid. *V*_lumen_ is the volume of the lumen (volume of distribution: 1 mL) under the conditions of the in situ closed-loop experiment.

### Statistical analysis

Data analysis was conducted using Microsoft Excel and Napp software (version 2.31 for Macintosh OS-X). All data are expressed as the mean of values obtained in at least three experiments with the standard error of the mean (SEM). Statistical analysis was conducted using GraphPad Prism 9 software. Multiple comparisons were done using one-way analysis of variance (ANOVA) followed by the Tukey–Kramer test, respectively.

## Data Availability

The datasets used and/or analyzed during the current study are available from the corresponding author on reasonable request.
